# A Case of Choledocholithiasis and Obstructive Jaundice With a Very High Serum Carbohydrate Antigen 19-9 (CA 19-9) Level: A Case Report and Review of Literature

**DOI:** 10.7759/cureus.32447

**Published:** 2022-12-12

**Authors:** Muhammad Ghallab, Hazem Abosheaishaa, Ishank Gupta, Salma Abdelmoteleb, Roger Stern

**Affiliations:** 1 Internal Medicine, Icahn School of Medicine at Mount Sinai, Queens Hospital Center, New York, USA; 2 Internal Medicine/Gastroenterology, Cairo University, Cairo, EGY; 3 Internal Medicine, Cairo University School of Medicine, Cairo, EGY; 4 Internal Medicine, Queens Hospital Center, New York, USA

**Keywords:** cholelithiasis, choledocholithiasis, obstructive jaundice, tumor markers, ca 19-9

## Abstract

Carbohydrate antigen 19-9 (CA 19-9) is a tumor marker used clinically to support the diagnosis and recurrence of pancreatic adenocarcinoma. CA 19-9 levels may be found to be elevated too in benign conditions, including hepatobiliary diseases such as choledocholithiasis, cholangitis, or cholestasis of any etiology. In this case, we are presenting a case of a 66-year-old man who has presented with diffuse abdominal pain and has been then found to have choledocholithiasis and obstructive jaundice treated with biliary sphincterotomy and balloon extraction. In the setting of extensive unclear gastroenterological surgeries, tumor markers were obtained, and the results revealed markedly elevated CA 19-9 with negative other tumor markers and a negative workup for malignancy. After reviewing the literature, we found that although CA 19-9 has been studied in diagnosing pancreatic and biliary malignancies, the serum level may also be elevated in many other benign conditions. As a result, the results must be individualized according to the clinical scenario of each patient.

## Introduction

Carbohydrate antigen 19-9 (CA 19-9) is a tumor marker, a sialylated lewis antigen, which is used clinically as a diagnostic indicator in supporting the diagnosis and recurrence of pancreatic adenocarcinoma, first identified in cultured human colonic carcinoma cells by Koprowski in 1979 [1]. It should be noted that CA 19-9 is a tumor-associated marker rather than a tumor-specific one. It can be found to be elevated in the pancreas, lung, colorectal, and liver malignancies, as well as benign conditions of the biliary system, pneumonia, and pleural effusions [2]. CA 19-9 levels may be elevated in hepatobiliary diseases such as choledocholithiasis, cholangitis, or cholestasis of any etiology and can be found elevated in various benign conditions. Thus, CA 19-9 is not known to be reliably used to distinguish between malignant and benign instances of extrahepatic jaundice. In this context, we present a case of a patient with a markedly elevated CA 19-9 level in a patient presenting with choledocholithiasis.

## Case presentation

A 66-year-old man with a past medical history of hypertension, hyperlipidemia, diabetes mellitus, obesity (BMI 30-39 kg/m^2^), exploratory laparotomy with bowel resection: Hartmann’s procedure (2013), reversal of Hartmann’s procedure with massive lysis of adhesions status post small bowel obstruction (SBO), followed by two admissions for SBOs (2017 and 2018) that were treated nonoperatively, presented to the emergency department with diffuse abdominal pain of three days duration. The patient described the pain as constant, non-radiating, 8/10 on a pain scale, worsened by sitting upright or lying on the right side and with consumption of food, and associated with nausea and non-bloody, non-bilious vomiting. The last bowel movement was the day before the presentation. Home medications included ramipril 10 mg, sitagliptin 50-1000 mg, and sodium polystyrene sulfonate 15 g/60 ml suspension. He had no known drug allergies. He denied any personal or family history of gastrointestinal cancers or cancers of other organ systems. He denied any fever, chills, or night sweats. He reported a 60-70 lbs weight loss over the past year with an otherwise unremarkable review of systems. He last consumed alcohol a year ago but denied a history of tobacco or illicit drug use. On initial management, his vitals showed a temperature of 36.7 Celsius, a heart rate of 75 beats/minute, blood pressure of 150/90, a respiratory rate of 18 breaths/minute, and oxygen saturation of 99%. His abdominal examination was remarkable for generalized abdominal tenderness that was more prominent in the periumbilical region without guarding, rigidity, or rebound tenderness.

**Figure 1:**
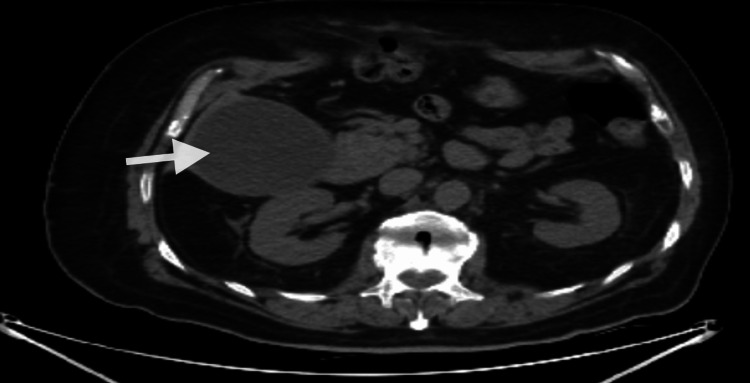
CT abdomen and pelvis without contrast showing markedly distended gallbladder (arrow) with minimal pericholecystic edema CT: computed tomography

Costovertebral tenderness was absent bilaterally. His head, eyes, ears, nose, and throat (HEENT), cardiovascular, pulmonary, and musculoskeletal examinations were unremarkable. Laboratory data demonstrated a white blood cell count of 9.11 K/mcl (baseline: 6.39-7.74 K/mcl), alkaline phosphatase 406 U/L, lipase 91 U/L (baseline: 34-39 U/L), total bilirubin 6.4 mg/dl, aspartate aminotransferase (AST) 112 U/L, alanine transaminase (ALT) 371 U/L. In the emergency department, he received an intravenous push of famotidine 20 mg, Zofran 4 mg, and 1 liter of normal saline. The computed tomography (CT) abdomen/pelvis without contrast shown in Figure 1 demonstrated a markedly distended gallbladder with minimal pericholecystic edema and dilation of intrahepatic and extrahepatic bile ducts with the common bile duct (CBD) measuring 1.9 cm. There was minimal peripancreatic edema suggestive of acute pancreatitis. An obstructive lesion involving the distal CBD was suspected. The gastroenterology service was consulted on day 1.

**Figure 2:**
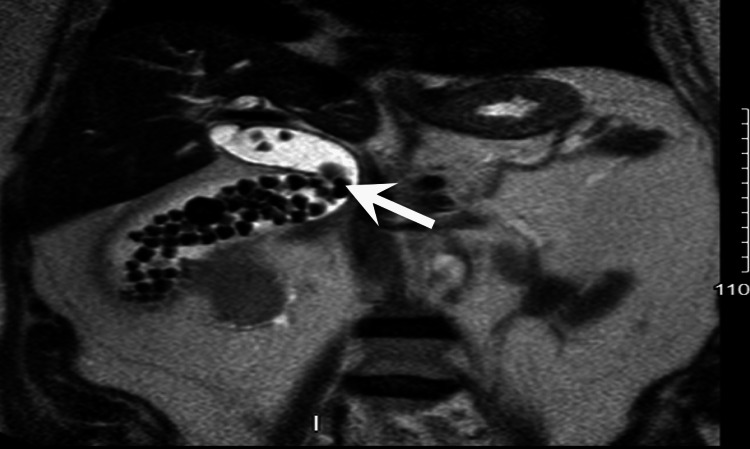
Magnetic resonance cholangiopancreatography shows a distended gallbladder (arrow) with numerous gallstones inside

**Figure 3:**
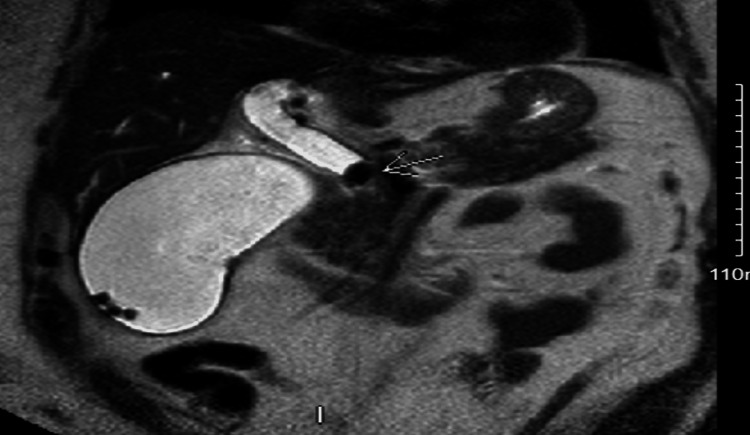
Magnetic resonance cholangiopancreatography shows a distended gallbladder with gallstones and shows dilated CBD blocked by a stone (arrow) CBD: common bile duct

The patient was scheduled for magnetic resonance cholangiopancreatography (MRCP), shown in Figures 2 and 3, which revealed a distended gallbladder with cholelithiasis and choledocholithiasis in the cystic duct and CBD. On day 2, the patient underwent endoscopic retrograde cholangiopancreatography (ERCP) with sphincterotomy for choledocholithiasis. The CBD was dilated during the procedure, and numerous stones were removed by biliary sphincterotomy and balloon extraction. Pus was drained from the duct by injecting contrast into the gallbladder without any clinical evidence of cholangitis.

 

Following ERCP, the patient had rising liver function tests and complained of right upper quadrant pain, possibly due to ampullary edema secondary to sphincterotomy and instrumentation. He was started on intravenous antibiotics with ciprofloxacin and Flagyl. On day 3, laboratory data demonstrated total bilirubin of 7.2 mg/dl, direct bilirubin of 6.2 mg/dl, alkaline phosphatase 411 U/L, AST 103 U/L, and ALT 312 U/L. Further laboratory investigations were unremarkable for hepatitis B virus, hepatitis C virus, human immunodeficiency virus, alpha-fetoprotein tumor marker, and carcinoembryonic antigen (CEA). However, the CA 19-9 was markedly elevated at 2192 U/ml. His hospital stay was extended to monitor liver function tests. He continued receiving intravenous antibiotics (ciprofloxacin and Flagyl) and intravenous Zofran for pain management. Throughout the hospital stay, he received gastrointestinal prophylaxis with pantoprazole 40 mg orally daily and prophylaxis for deep vein thrombosis with low molecular weight heparin. Sitagliptin was discontinued in the setting of pancreatitis, and he has been prescribed metformin in light of well-controlled type 2 diabetes mellitus (H1ABc 5.8). He did not receive any blood transfusions during this hospitalization. On day 6, liver function tests trended down, and he was able to tolerate diet and reported only mild right upper quadrant abdominal pain. He was discharged on a six-day prescription of oral metronidazole and ciprofloxacin with a scheduled outpatient follow-up.

## Discussion

CA 19-9 is made mainly by the pancreatic ductal cells and the biliary tract. It can also be made by epithelial cells of the stomach and salivary glands. As mentioned above, CA 19-9 is mainly associated with pancreatic cancer, specifically pancreatic adenocarcinoma. However, it is also elevated in various benign, malignant, gastrointestinal, and extra-gastrointestinal diseases [3-5]. CA 19-9 is the most specific and sensitive marker used in the diagnosis of pancreatic malignancies. In the absence of jaundice and at levels greater than 1000 U/ml, the specificity is almost 100% at levels above 1000 U/ml without clinical jaundice. However, more than 1000 U/ml levels are uncommon for non-malignant diseases [6]. The reason for the high CA 19-9 in malignancies can be explained by its excessive secretion by the malignant cells. However, the exact reason for its increased level in non-malignant conditions is still unclear. It’s hypothesized that it could be due to biliary tract obstruction's leakage into the blood. Another hypothesis postulated that the inflammatory cytokines released in the setting of sepsis due to cholangitis might contribute to the high level of CA 19-9 [7].

Benign diseases with highly elevated CA 19-9 are relatively rare. This literature review addresses some cases with elevated CA 19-9 without malignancy. In 2002, Katsanos et al. [8] reported four examples of exceptionally high CA 19-9 levels in benign biliary tract illnesses. The first patient was a 77-year-old male who presented with jaundice and choledocholithiasis. The CA 19-9 level was 98,628 IU/ml. The second patient was a 63-year-old male patient with autoimmune cholangitis and a CA 19-9 level of 250 IU/ml. In the third instance, acute cholangitis developed in a 74-year-old male patient who had choledocholithiasis and cholelithiasis. The CA 19-9 levels peaked at 14,950 IU/ml. In the fourth instance, a 73-year-old man developed biliary colic and jaundice and had undergone open cholecystectomy with a brief 100-fold increase in CA 19-9 (2230 IU/ml). Trompetas et al. [9] reported an 81-year-old man with gallbladder agenesis who presented with choledocholithiasis and obstructive jaundice. The CA 19-9 serum level was (2765 U/ml). After CBD decompression, all symptoms resolved, and the CA 19-9 level recovered to normal.

Additionally, Sanchez et al. [10] reported a patient with markedly elevated CA 19-9 and imaging suggestive of malignancy. However, Mirizzi syndrome was identified during laparotomy with no evidence of malignancy. This case indicates that it’s important to read and interpret the CA 19-9 level cautiously in the presence of biliary obstruction. Marcouizos et al. [11] also reported a case of a 79-year-old woman who presented with right upper quadrant pain, fever, chills, and jaundice. She was found to have obstructive jaundice and underwent surgery. The CA 19-9 initial level was 99.070 U/ml, then dropped to 970 U/ml two weeks after surgery before returning to the normal range (31 U/ml) two months later. In 2010, Korkmaz et al. [12] documented a case of cholelithiasis and choledocholithiasis with acute cholangitis that had extremely high serum levels of CA 19-9 (9586 IU/ml), which decreased sharply after therapy. Canete-Ruiz et al. [13] reported a case of a 69-year-old patient who presented with severe abdominal pain and deep jaundice. The patient was diagnosed with choledocholithiasis with dilated bile ducts with significant elevation of CA 19-9 to >12,000 IU/ml. He underwent ERCP with stone extraction. Gradually, the patient's CA 19-9 level decreased to 366.4 IU/ml at the time of discharge.

The clinical characteristics and underlying reasons for CA 19-9 elevation in the absence of malignant or pancreatobiliary disease were examined in a retrospective study by Kim et al. [5]. Following an examination of the patient's medical records, it was discovered that the CA 19-9 elevation was unrelated to malignancy after follow-up. From 192 patients that were included in the study, the causes of the CA 19-9 increase were identified in 147 patients, while 45 patients had unknown origins. The origin of identified causes was hepatic, lung, gynecologic, endocrine, and splenic diseases. Thirty-five of the 45 patients with an unclear reason had their CA 19-9 levels return to normal, and ten had persistently increased CA 19-9 [5]. Haring et al. reported an interesting case of an 84-year-old male who presented with unexplained icterus with imaging suggestive of pancreaticobiliary malignancy. The CA 19-9 concentration was 4240 IU/ml. ERCP was subsequently done and revealed obstructive choledocholithiasis. The patient then underwent laparoscopic cholecystectomy, and the analysis of the gallbladder's histology led to the diagnosis of xanthogranulomatous cholecystitis. After surgery, CA 19-9 levels dropped, and the patient quickly recovered [14]. Interestingly, Tan et al. reported a biliary adenoma case in a 74-year-old Chinese man after CBD exploration. The initial presentation was fever and painless obstructive jaundice with significantly raised CA 19-9, but his condition significantly improved after open CBD excision and Roux-en-Y hepaticojejunostomy [15]. According to the literature that is currently accessible, increased CA 19-9 has also been seen in a number of cases of mild hydronephrosis and a few benign renal disorders [16-18].

## Conclusions

In conclusion, CA 19-9 is a tumor marker that has received substantial research and widespread use for the diagnosis and prognosis of pancreaticobiliary cancers. Clinicians should exercise caution when interpreting CA 19-9 results because levels may be significantly influenced and elevated in many other benign diseases, making it difficult to distinguish between benign and malignant causes of elevated CA 19-9. These results must be viewed in the context of the clinical scenario of each patient.
